# Additive toxicity arising from combined use of immune checkpoint inhibitors and tyrosine kinase inhibitors in patients with renal or endometrial carcinoma: Protocol for a rapid systematic review

**DOI:** 10.1016/j.mex.2024.102730

**Published:** 2024-04-26

**Authors:** Shannon E. Kelly, Xiaoqin Wang, Shu-Ching Hsieh, Aws Abdul-Wahid, Melanie Derry, Becky Skidmore, George A. Wells

**Affiliations:** a Cardiovascular Research Methods Centre, University of Ottawa Heart Institute, Ottawa, Ontario, Canada; b School of Epidemiology and Public Health, University of Ottawa, Ottawa, Ontario, Canada; c Bureau of Biologics, Radiopharmaceuticals and Self-Care Products, Marketed Health Products Directorate, Health Products and Food Branch, Health Canada, Ottawa, Ontario, Canada; d Independent Information Scientist, Ottawa, Ontario, Canada

**Keywords:** Immune checkpoint inhibitors, Tyrosine kinase inhibitors, Safety, Combined therapy, Systematic review

## Abstract

The combined use of immune checkpoint inhibitors and tyrosine kinase inhibitors (ICI/TKI) is an effective treatment strategy for some cancers. A better understanding of the potential additive toxicity for ICI/TKI combinations is needed to inform patient and provider treatment decisions. We aim to evaluate the safety of ICI/TKI combinations for individuals with renal cell or endometrial carcinoma. This rapid systematic review (SR) protocol follows PRISMA guidelines. A systematic search will be designed, peer reviewed and executed by experienced information specialists (Cochrane Central, MEDLINE, Embase) to identify published SRs and primary studies published since the most recent SR search. Randomized, quasi- or non-randomized controlled trials and comparative cohort studies are eligible if they compare ICI/TKI combinations to monotherapy or standard of care in participants with renal cell or endometrial carcinoma. The primary outcome is grade ≥ 3 treatment-related adverse-effects. Studies will be screened, selected, extracted and assessed for risk of bias by a single reviewer and checked completely by a second. Where feasible and appropriate, we will pool studies separately by design and indication using meta-analysis and test robustness of effects using prespecified subgroup and sensitivity analyses. Results will be summarized descriptively and presented in tables and figures. (PROSPERO ID: CRD42023416388).

• This will be a comprehensive systematic review of the additive toxicity arising from the combined use of ICI/TKIs in patients with renal-cell or endometrial carcinoma.
• We will consider treatment-related, treatment-emergent adverse events (Grade 3 or higher).
• Identified safety profile may be used to inform patient or provider treatment decisions.

## Graphical abstract

This is special type of abstract that is so short and could be inserted after main abstract of article, as a blurb or inserted as annotations into a Table of contents

**Figure:**
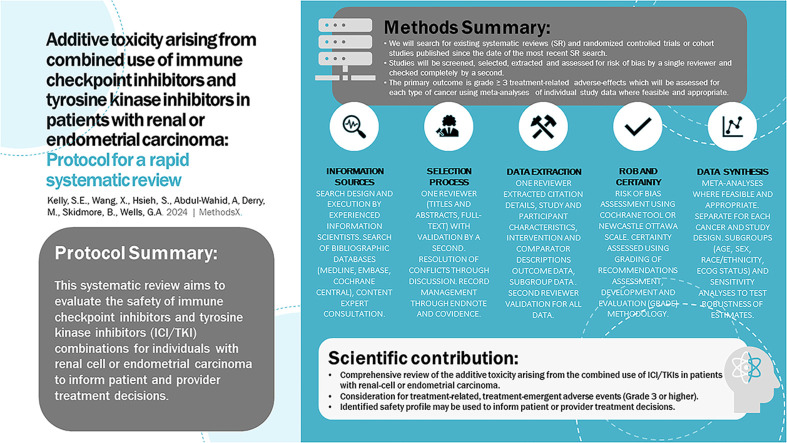

Specifications table

**Table:** 

Subject area:	Medicine and Dentistry
More specific subject area:	Cancer: Renal cell carcinoma, Endometrial carcinoma
Name of your protocol:	Additive toxicity arising from combined use of immune checkpoint inhibitors and tyrosine kinase inhibitors in patients with renal or endometrial carcinoma: Protocol for a rapid systematic review
Reagents/tools:	Not applicable
Experimental design:	Not applicable/Systematic review protocol
Trial registration:	PROSPERO ID: CRD42023416388
Ethics:	No ethics approval is required as all data reviewed and collected will be publicly available. No data will be collected using social media,
Value of the Protocol:	• We aim to comprehensively evaluate the additive toxicity of combined TKI and ICI drugs by focusing on key harms in populations where use in practice is common (renal cell carcinoma and endometrial carcinoma).
	• We will use systematic methods in accordance with the methods of the Cochrane Adverse Events and Rapid Reviews Methods Groups and results will be reported as recommended by the Preferred Reporting Items for Systematic Reviews and Meta-Analyses (PRISMA) Harms Statement.
	• A rigorous search strategy will be used that will be designed an executed by an information scientist experienced with drug safety reviews and peer-reviewed by a second experienced information scientist in accordance with the Peer Review of Electronic Search Strategies (PRESS) statement.

## Description of protocol

### Methods

This is a protocol for a rapid systematic review to assess the additive toxicity of combined use of ICIs and TKIs compared to either ICI or TKI monotherapy or standard of care for individuals with renal cell or endometrial carcinoma. The protocol was drafted using the Preferred Reporting Items for Systematic Reviews and Meta-analysis Protocols (PRISMA-P) [1,2] and through the International Prospective Register of Systematic Reviews (currently under assessment in PROSPERO, ID 416388) [3,4].

The systematic review will use a rapid approach which is an accepted mythology for knowledge synthesis in which components of the systematic review process are omitted or tailored with the aim to produce findings in a timely manner [5], [6], [7], [8]. Any tailoring or deviations from the Preferred Reporting Items for Systematic Reviews and Meta-Analyses (PRISMA) criteria will be declared [9]. This review will be conducted in line with published guidance from the Cochrane Rapid Review and Adverse Effects Methods Groups [10,11] and reported using the PRISMA and PRISMA harms checklist [9,12].

The primary research question guiding the rapid systematic review aims to assess treatment-related adverse effects:

1. What is the safety (according to grade ≥ 3 treatment-related adverse-effects) of combined use of ICI and of TKIs compared to ICI or TKI alone, or standard of care for individuals with (a) renal cell carcinoma or (b) endometrial carcinoma.

The secondary research questions aim to assess treatment-emergent adverse effects, treatment discontinuation due to adverse effects, and whether safety outcomes vary by important subgroups:

2. What is the safety (according to grade ≥ 3 treatment-emergent adverse-effects) of combined use of ICI and of TKIs compared to ICI or TKI alone, or standard of care for individuals with (a) renal cell carcinoma or (b) endometrial carcinoma.
3. What is the safety (according to treatment discontinuation due to treatment-related and treatment-emergent adverse effects) of combined use of ICI and of TKIs compared to ICI or TKI alone, or standard of care for individuals with (a) renal cell carcinoma or (b) endometrial carcinoma.
4. Does the safety (according to grade ≥ 3 treatment-related adverse-effects) of combined use of ICI and of TKIs vary in important subgroups (sex, age, race/ethnicity, Eastern Cooperative Oncology Group (ECOG) performance status scale, baseline liver or kidney status) of individuals with (a) renal cell carcinoma or (b) endometrial carcinoma compared to ICI or TKI alone, or standard of care.

### Eligibility criteria

We used a population, intervention, comparator, outcome and study design (PICOS) framework to define eligibility criteria (Table 1) [13]. We will include primary studies that compare adverse effects of any ICI/TKI combination for participants with any stage of renal cell or endometrial carcinoma, regardless of treatment history. We will include experimental studies (randomized clinical trials [RCTs], quasi-RCTs, non-RCTs) and comparative cohort studies. The rationale for including other study designs in addition to RCTs is that substantial RCT evidence may not exist for these combination therapies, especially in populations with endometrial carcinoma. Also, the assessment of harms can require relatively large sample sizes to be able to identify adverse events. While RCTs may offer advantages in that they may have lower risk of bias, they may not be powered statistically to adequately evaluate uncommon or rare safety outcomes due to Type II (i.e., false negative) error [14]. Given that this review aims to comprehensively assess harms, consideration of comparative cohort studies is appropriate. We will exclude studies relatively reporting mixed cancer populations (e.g., multi-cohort trials), unless it is possible to extract the data separately for renal cell and/or endometrial carcinoma participants.

**Table 1:** Population, intervention, comparator, outcome, study design (PICOS) framework.

**PICOS elements**	Renal cell carcinoma	Endometrial carcinoma
**Population**	Individuals of any age diagnosed with renal cell carcinoma	Individuals of any age diagnosed with endometrial carcinoma
**Intervention**	Any combined use of immune checkpoint inhibitors and of tyrosine kinase inhibitors^a^	
**Comparators**	• immune checkpoint inhibitor monotherapy • tyrosine kinase inhibitor monotherapy • standard of care (e.g., sinitinib)	
**Primary outcome**	Grade ≥ 3 treatment-related adverse events	
**Secondary outcomes**	• Grade ≥ 3 treatment-emergent adverse events • Treatment discontinuation due treatment-related and treatment-emergent adverse events	
**Study designs**	• Randomized controlled trials • Comparative cohort studies	

a Regardless of regulatory status.

Studies not reporting outcomes of interest will be included and checked against other included records and monographs for associated publications or linked data. Primary studies reported in abstracts or letters to the editor are eligible for inclusion. We will not place any restrictions on the language of publication. Studies in-progress, protocols, registry records marked completed but without results, and those not reporting eligible outcomes of interest will be cited in the final report but will not undergo data extraction, risk of bias assessment or be included in results beyond the description of the search findings. Studies that cannot be translated within the reasonable time limits of the review will be reported as records in-process.

### Information sources and search strategy

We will conduct a comprehensive literature search using a strategy drafted by an experienced research librarian. A second experienced research librarian will peer-review the MEDLINE search strategy using the Peer Review of Electronic Search Strategies (PRESS) checklist [15]. The final electronic search strategy will incorporate feedback from the peer-review process Using the multifile option and deduplication tool available on the Ovid platform, we will search Ovid MEDLINE® ALL, Embase and the Cochrane Database of Systematic Reviews (CDSR) for the review search. For the primary studies, we will substitute Cochrane CENTRAL for CDSR. The search strategy will employ both controlled vocabulary (e.g., “Immune Checkpoint Inhibitors”, “Drug Therapy, Combination”, “Endometrial Neoplasms”) and key words (e.g., TKI, polytherapy, renal cell cancer), and will combine concepts pertaining to ICI and TKI drugs, ‘combination drug therapy’ and the populations of interest. A draft search for MEDLINE is included in Appendix 1.

The search will be executed sequentially with the initial electronic database search aimed at locating systematic reviews of eligible primary studies. A study design filter derived from the CADTH filter for systematic reviews will be applied [16]. Following this, a top-up search will be executed to locate primary studies published after the search date of the most recent systematic review for (a) renal cell carcinoma or (b) endometrial carcinoma. In the event no systematic reviews are located for endometrial carcinoma, a five-year date-limited search for primary studies will be executed. In searches for primary studies, a safety filter will be used to catch relevant terminology found in a record title, abstract, keywords or indexing [17]. For all searches, results will be downloaded and deduplicated using EndNote version 9.3.3 (Clarivate Analytics).

No additional limitations will be placed on the search (e.g., language restrictions) although where possible, animal-only records and opinion pieces will be removed. The electronic database search will be supplemented by searching clinicaltrials.gov for completed registrations and from manufacturer product monographs.

### Record selection

An eligibility criteria screening form will be pilot-tested on the same 50 titles and abstracts prior to the start of formal record selection. All screening will be conducted using DistillerSR systematic review software (DistillerSR. Version 2.39.0. Evidence Partners; 2022).

For screening of systematic reviews including potentially relevant records: One reviewer will screen each title and abstract for inclusion along with any potentially relevant full-text records. A second reviewer will confirm inclusions. Discrepancies will be resolved by discussion or the involvement of a third reviewer. One reviewer will extract potentially relevant primary study citations from each included systematic review and assess the full-text for eligibility using the population, intervention, comparator and study design. A second reviewer will confirm all inclusion decisions and any discrepancies will be resolved through consensus or by a third reviewer.

For screening of potentially relevant primary study records: One reviewer will screen each title and abstract for inclusion. Subsequently, two reviewers will independently screen any full-text records for inclusion. All identified product monographs and clinical registry records will be screened by one reviewer and all decisions for inclusion or exclusion will be confirmed by a second reviewer. Inter-rater discrepancies will be resolved by discussion or a third reviewer.

### Data collection

Data extraction will be conducted by a single reviewer with complete independent checking by a second reviewer. Discrepancies will be discussed and resolved with a third reviewer if necessary. Data for renal cell and endometrial carcinoma studies will be extracted into separate but similarly standardized data forms following a pilot.

The following data will be extracted from included primary studies: general information on the citation, publication type, study characteristics including but not limited to: design, hypothesis, unit of allocation, start/end date, duration of participation/follow-up, number of centres, participant recruitment and flow through the study (including lost to follow-up, crossover etc.), participant characteristics (population description, setting, comorbidities, cancer stage, sociodemographic and baseline characteristics, including equity variables described by PROGRESS- Plus) [18], and details about the interventions and study groups (including interventions and comparator descriptions, dose, frequency, timing, delivery, providers, co-interventions, compliance duration of treatment, etc.). Data will be collected for treatments and controls separately where possible.

Data will be collected for outcomes of interest (number with an event, total in group) for each time point and subgroup reported. Missing data will be noted and text for and associated reason. For all outcomes we will endeavor to collect study-specific outcome definitions, how the outcome was ascertained (e.g., patient report, active search, investigator report, database), and over what time period. Any adjudication as part of study conduct will be noted with details about who performed any adjudication and under what study conditions. The adverse event grade applied by study authors will be used when reported, else, reported harms will be categorized by grade based using definitions from the National Cancer Institute's Common Terminology Criteria for Adverse Events (CTCAE, v5.0) [19]. Study funding sources and declared conflicts will also be recorded.

Data will be collected for systematic reviews used to identify primary studies, including citation, PICOS, search dates, number of studies included and author conclusions. Summary tables will be used to present the population and study characteristics.

### Data management and sharing

Data management processes will follow guidance from the Digital Research Alliance of Canada for research data management systematic reviews and be documented alongside review findings [20]. The anticipated types of data used in this review will be literature database records (e.g., .RIS, BibTex, .txt); full-text of articles (PDF); quantitative and qualitative data extracted from individual studies (.xlsx), and; documents describing review processes and methods (.docx, PDF). Files will be named and stored using structured, project-specific and version-controlled naming conventions. Secure storage is not required as no individual patient or otherwise sensitive data will be considered. Anticipated data sharing will include complete search strategies for all databases, data extraction templates, summary and analyzed data in tables and figures.

### Risk of bias

Risk of bias in the included trials will be appraised using the Cochrane risk of bias tool for RCTs (v.1.0) and the Newcastle Ottawa Scale for comparative cohort studies by one reviewer, with checking by a second reviewer [21,22]. Discrepancies will be discussed and resolved with a third reviewer if necessary.

### Synthesis and analysis

A descriptive summary of study results will be presented along with evidence summary tables. Studies will be pooled separately by population (renal cell or endometrial carcinoma), study design (randomized, quasi, cohort) and comparator (ICI monotherapy, TKI monotherapy, standard of care). We will make the following comparisons in the analysis.

• For renal cell carcinoma:
  ○ any combination of ICI and a TKI versus ICI monotherapy
  ○ any combination of ICI and a TKI versus TKI monotherapy
  ○ any combination of ICI and a TKI versus standard of care
• For endometrial carcinoma:
  ○ any combination of ICI and a TKI versus ICI monotherapy
  ○ any combination of ICI and a TKI versus TKI monotherapy
  ○ any combination of ICI and a TKI versus standard of care

Comparisons of ICI/TKI combinations to monotherapy with an ICI or TKI will be the primary comparison. Prior to any synthesis, statistical, clinical, and methodological heterogeneity will be examined. Where data from RCTs are sufficient and analyses are feasible, pairwise meta-analysis will be conducted using the relative risk of the dichotomous outcomes to combine studies addressing the same outcome and treatment comparison. A random-effects model will be employed, as we expect methodological and clinical heterogeneity across the included studies. Between- study heterogeneity (τ2) will be examined using the restricted maximum likelihood method, and quantified using the I^2^ statistic [23,24]. If heterogeneity is extensive (e.g., I^2^ ≥ 75 %), clinically relevant subgroup and meta-regression techniques will be considered.

For comparative cohort studies, random-effects pairwise meta-analysis will be conducted using the adjusted effect estimates (relative risk or equivalent) of the dichotomous outcomes to combine studies addressing the same outcome and treatment comparison. If adjusted estimates are not available, raw data will be considered for the effect estimates and sensitivity analyses conducted. Methods for analyses involving studies with zero events in at least one or all study groups will follow approaches proposed for zero event classification [25,26]. If necessary, we will impute missing data (e.g., measures of variance). The analyses of drug combinations will be conducted using accepted methodological approaches [27,28].

Publication bias will be assessed using funnel plots for outcomes including at least 10 studies to explore asymmetry that might be explained by clinical, statistical, and methodological heterogeneity. RevMan 5 software (https://training.cochrane.org/online-learning/core-software/revman) will be used to conduct all pairwise meta-analyses. Results will be presented in tables and figures (forest plots).

### Subgroups

It is important to understand whether there are any subgroups of cancer patients who are more at risk for toxicity with combination therapy of ICIs and TKIs. Where data are available and analyses are feasible, we will consider clinically-important subgroups based on age, sex, race/ethnicity, ECOG Performance Status Scale, and baseline liver and/or kidney status by cancer indication. We are interested in how treatment harms vary in pediatric (<18 years), adult and elderly (<75 years) populations, and by biological sex (men/women). We will follow updated guidance for the considering and reporting of demographic subgroups based on race/ethnicity in medical and science journals [29]. Race and ethnicity categories will be collected based on the specific categories reported by the primary studies recognizing that categories may differ across studies based on how the information was collected, the source of the classification (e.g., self-report, investigator assigned, database, survey etc.).

The ECOG Performance Status Scale is utilized to track changes in a patient's individual level of functioning as a result of treatment during a cancer research study and considers, for example, ability to care for oneself, activities of daily living, and physical ability (Table 2) [30]. Renal function will be considered based on reported glomerular filtration rate (GFR; normal >60, kidney disease <60, kidney failure >15), urine albumin to creatinine ratio (uACR; normal to mildly increased < 30 mg/g, moderately increased 30 to 299 mg/g, severely increased ≥ 300 mg/g), or data for patients enrolled with chronic kidney disease. Liver function will be considered based on reported indicators such as serum bilirubin or albumin level elevation will be used, or data for patients enrolled with chronic liver disorders, hepatitis or liver-related cholestasis. We will use the PROGRESS-Plus framework to identify how outcomes may vary according to equity-related variables with consideration to populations experiencing inequities and those typically underrepresented in clinical studies. We will present results of any equity-related subgroup analyses where feasible and discuss all findings with an equity lens considering generalizability [18]. Results will be presented in tables and figures (forest plots).

**Table 2:** ECOG Performance Status Scale [30].

GRADE	ECOG PERFORMANCE STATUS
**0**	Fully active, able to carry on all pre-disease performance without restriction
**1**	Restricted in physically strenuous activity but ambulatory and able to carry out work of a light or sedentary nature, e.g., light house work, office work
**2**	Ambulatory and capable of all selfcare but unable to carry out any work activities; up and about more than 50 % of waking hours
**3**	Capable of only limited selfcare; confined to bed or chair more than 50 % of waking hours
**4**	Completely disabled; cannot carry on any selfcare; totally confined to bed or chair
**5**	Dead

### Sensitivity analyses

We will perform sensitivity analyses to investigate the robustness of the meta-analysis results for the primary outcome and comparison [24]. While additional issues suitable for sensitivity analyses may be identified during the review process, we will pre-specify sensitivity analyses considering any inferred or unclear outcome data, fixed effects for meta-analysis, excluding abstracts, and using alternative methods for the analyses of zero events (36). Results will be presented in tables and figures (forest plots).

### Certainty of evidence

For RCTs, one reviewer will construct evidence profiles of certainty for each outcome and comparison, using GRADEPro (GRADEpro GDT [Software]. McMaster University and Evidence Prime, 2024. Available from gradepro.org). A second reviewer will check all tables, and they will be vetted by the review team and a content expert. The certainty of the evidence will be determined by the risk of bias across studies, indirectness, and imprecision Owing to the rapid review approach, reviewers will not consider other parameters. We will use the most recent Grading of Recommendations Assessment, Development and Evaluation (GRADE) methodology to decide on the certainty of the body of evidence from RCTs, which recommends using the judgment of high certainty of evidence at baseline and downgrading due to risk of bias, imprecision, or indirectness of RCTs [31]. Important clinical differences and optimal information size (OIS; 1000 participants) will be confirmed in advance through consult with content experts and used to inform judgements about precision. Downgrading 3 levels for precision will be considered when the number of study participants for an outcome is less than 30 % of the OIS [32,33]. Plain language statements determined using the ratings for the certainty of evidence will be presented with the ratings [34].

### Ethics and dissemination

Results from the rapid systematic review will be disseminated through a peer-reviewed and open publication and stakeholder meetings, and potentially through relevant symposia. This study does not require ethics approval as the rapid systematic review methodology consists of reviewing and collecting data from publicly available materials.

## Funding

This systematic review was funded by the Canadian Institutes for Health Research, Drug Safety and Effectiveness Network (CIHR/DSEN).

## CRediT authorship contribution statement

**Shannon E. Kelly:** Conceptualization, Methodology, Funding acquisition, Writing – original draft. **Xiaoqin Wang:** Writing – review & editing. **Shu-Ching Hsieh:** Writing – review & editing. **Aws Abdul-Wahid:** Conceptualization, Writing – original draft, Writing – review & editing. **Melanie Derry:** Conceptualization, Writing – original draft, Writing – review & editing. **Becky Skidmore:** Methodology, Writing – original draft, Writing – review & editing. **George A. Wells:** Conceptualization, Methodology, Funding acquisition, Writing – review & editing.

## Declaration of competing interest

The authors declare that they have no known competing financial interests or personal relationships that could have appeared to influence the work reported in this paper.

## Data Availability

No data was used for the research described in the article.

## Supplementary material *and/or* additional information

### Background

Immune check point inhibitors (ICIs) are monoclonal antibodies that target and block the activation of immune inhibitory checkpoint pathways within the tumor microenvironment [35]. This leads to robust T-cell activation and expansion, as well as initiation of anti-tumor immune responses [36]. Given the ubiquitous expression of immune-checkpoints by many tumor types, ICIs have been authorized for the treatment of many cancers, including but not limited to renal cell and endometrial carcinoma [37,38], and their use has become integrated as part of the care offered to cancer patients.

One of the current strategies of ICI use has been their combination with tyrosine kinase inhibitors (TKIs). TKIs are a group of pharmaceutical anti-cancer drugs that disrupt the signal transduction pathways of protein kinases through different modes of inhibition [39,40]. This strategy has proven to be effective against various cancers in clinical trials recent years [41], [42], [43], [44], and regulators have authorized the use of ICIs with TKIs in the treatment of both renal cell and endometrial carcinomas [37,42].

However, activation of the immune system by ICIs can lead to immune-related adverse events (irAEs) [45], and TKIs are related to several adverse reactions including diarrhoea, hypertension, fatigue, hypothyroidism, palmar–plantar erythrodysesthesia syndrome (hand–foot syndrome), and gastrointestinal symptoms (nausea, loss of appetite, stomatitis) [46] and cutaneous adverse drug reactions as the most common severe adverse effects [39]. In addition, the adverse reactions appear to numerically add up when using TKI/ICI combination therapies [46].

The potential for additive toxicity arising from the combination of ICIs and TKIs represents a serious impact in cancer patients who are medically vulnerable and often without access to alternate treatments. Given the availability of a substantial body of published information (from clinical trials as well as other sources), there is now available data to consider a systematic review and meta-analysis to better characterize this safety concern. The primary objective of this rapid systematic review is to assess the additive toxicity of combined use of ICIs and TKIs compared to either ICI or TKI monotherapy or standard of care for individuals with renal cell or endometrial carcinoma. Results will address knowledge gaps for a variety of knowledge users, including payers or funders, regulators who are interested in monitoring marketed drug products to inform regulatory actions and providers, patients and caregivers who want to make informed treatment decisions.

## Appendix: Search strategy for medline

TKIs/Immune Checkpoint Inhibitors – Endometrial/Renal Cell Carcinoma

Systematic Reviews

Scoping

2023 Mar 9

MEDLINE

Database: Ovid MEDLINE(R) ALL <1946 to March 08, 2023>

Search Strategy:

1. Immune Checkpoint Inhibitors/ (7871)
2. ((immune checkpoint or CTLA-4 or Cytotoxic T-Lymphocyte-Associated Protein 4 or PD-1 or PD-1-PD-L1 or PD-L1 or Programmed Cell Death Protein 1 or Programmed Death-Ligand 1) adj3 (inhibition or inhibitor? or blocker? or blockade?)).tw,kw,kf. (34,636)
3. ((ICI or ICIs) adj5 immun*).tw,kw,kf. (7950)
4. (atezolizumab or mpdl 3280 or mpdl3280 or mpdl3280a or rg 7446 or rg7446 or ro 5,541,267 or ro5541267 or tecentriq$2 or tecntriq$2 or 1,380,723–44–3 or 52CMI0WC3Y).tw,kw,kf,ot,hw,rn,nm. (2813)
5. (avelumab or bavencio$2 or "msb 0010682″ or msb0010682 or msb 0010718c or msb0010718c or msb 10,682 or msb10682 or msb 10718c or msb10718c or "pf 06834635″ or pf06834635 or pf 6,834,635 or pf6834635 or 1,537,032–82–8 or KXG2PJ551I).tw,kw,kf,ot,hw,rn,nm. (877)
6. Nivolumab/ (4920)
7. (nivolumab or bms 936,558 or bms936558 or cmab 819 or cmab819 or mdx 1106 or mdx1106 or ono 4538 or ono4538 or opdivo$2 or 946,414–94–4 or 31YO63LBSN).tw,kw,kf,ot,hw,rn,nm. (9173)
8. (pembrolizumab or keytruda$2 or Merck 3475 or MK-3475 or sch-900,475 or sch900475 or lambrolizumab$2 or 1,374,853–91–4 or DPT0O3T46P4).tw,kw,kf,ot,hw,rn,nm. (8421)
9. or/1–8 [**IMMUNE CHECKPOINT INHIBITORS, GENERAL AND SPECIFIC**] (44,574)
10. Protein Kinase Inhibitors/ (57,129)
11. Tyrosine Protein Kinase Inhibitors/ (103)
12. (tyrosine adj2 kinase inhibitor?).tw,kw,kf. (36,414)
13. (TKI or TKIs).ti,kw,kf. (3579)
14. ((TKI or TKIs) adj10 (tyrosine or protein or kinase or inhibit*)).ab. (12,691)
15. Axitinib/ (702)
16. (axitinib or "ag 013736″ or ag013736 or ag13736 or ar 14,034 or ar14034 or inlyta$2 or 319,460–85–0 or C9LVQ0YUXG).tw,kw,kf,ot,hw,rn,nm. (1445)
17. (cabozantinib or bms 907,351 or bms907351 or cabometyx or cometriq or xl 184 or xl184 or 849,217–68–1 or 1C39JW444G).tw,kw,kf,ot,hw,rn,nm. (1526)
18. (lenvatinib or "aiv 007″ or aiv007 or e 7080 or er 203,492–00 or er203492–00 or kisplyx$2 or lenvima$2 or mk 7902 or mk7902 or 417,716–92–8 or 942,407–57–0 or EE083865G2).tw,kw,kf,ot,hw,rn,nm. (1928)
19. (pazopanib or armala or gw 786,034? or gw786034? or sb 710,468? or sb710468? or votrient).tw,kw,kf,ot,hw,rn,nm. (2274)
20. Sunitinib/ (4097)
21. (sunitinib or gb 102 or gb102 or pha 2909040ad or pha 290940ad or pha2909040ad or pha290940ad or pno 290,940 or pnu290940 or "su 010398″ or "su 011248″ or su 10,398 or su 11,248 or su010398 or su011248 or su10398 or su11248 or sutent or 557,795–19–4 or 326,914–13–0 or V99T50803M).tw,kw,kf,ot,hw,rn,nm. (7361)
22. or/10–21 [**TKIs, GENERAL AND SPECIFIC**] (88,563)
23. 9 and 22 [**IMMUNE CHECKPOINT INHIBITORS, TKIs**] (3066)
24. Drug Therapy, Combination/ (174,442)
25. ((combin* or multi*) adj3 (drug ther* or drug treatment* or chemother* or chemo-ther* or immunotherap* or immuno-therap*)).tw,kw,kf. (66,630)
26. ((2-drug? or two-drug? or 3-drug? or three drug? or triple drug? or triplet or 4-drug? or four-drug? or quadruple drug? or multi-agent? or multiagent? or multi-drug? or multidrug? or multiple agent? or multiple drug?) adj3 (treatment* or chemother* or chemo-ther* or immunotherap* or immuno-therap* or therap*)).tw,kw,kf. (14,898)
27. (polytherap* or polychemotherap* or poly-therap* or poly-chemotherap* or poly-chemo-therap* or polychemo-therap* or polyimmunotherap* or poly-immuno-therap* or polyimmuno-therap*).tw,kw,kf. (5354)
28. (immune-based adj3 combination?).tw,kw,kf. (84)
29. or/24–28 [**COMBINATION DRUG THERAPY**] (248,545)
30. (9 or 22) and 29 [**IMMUNE CHECKPOINT INHIBITORS, TKIs - COMBINATION DRUG THERAPY - Pt 1**] (7340)
31. ((immuno* or immune checkpoint or ICI or ICIs) adj5 (TKI or TKIs or tyrosine)).tw,kw,kf. **[IMMUNE CHECKPOINT INHIBITORS, TKIs - COMBINATION DRUG THERAPY - Pt 2**] (9025)
32. 30 or 31 [**IMMUNE CHECKPOINT INHIBITORS, TKIs - COMBINATION DRUG THERAPY - Pts 1 and 2**] (16,179)
33. 23 or 32 [**IMMUNE CHECKPOINT INHIBITORS, TKIs, COMBINATION DRUG THERAPY**] (18,328)
34. exp Endometrial Neoplasms/ (25,425)
35. ((endometri* or uterine papillary serous) adj3 (carcinoma* or adeno-carcinoma* or malignan* or metasta* or neoplasia* or neoplasm* or sarcoma* or tumour* or tumor*)).tw,kw,kf. (21,944)
36. Kidney Neoplasms/ (78,103)
37. Carcinoma, Renal Cell/ (39,425)
38. ((collecting duct or hypernephroid or hyper-nephroid or kidney or nephroid* or renal) adj3 (carcinoma* or adeno-carcinoma* or malignan* or metasta* or neoplasia* or neoplasm* or sarcoma* or tumour* or tumor*)).tw,kw,kf. (75,436)
39. (grawitz* tumo?r or hypernephroma* or hyper-nephroma* or mRCC).tw,kw,kf. (3987)
40. or/34–39 [**ENDOMETRIAL, RENAL CANCER**] (140,501)
41. 33 and 40 [**IMMUNE CHECKPOINT INHIBITORS, TKIs, COMBINATION DRUG THERAPY - ENDOMETRIAL, RENAL CANCER**] (1430)
42. exp Animals/ not Humans/ (5,100,299)
43. 41 not 42 [**ANIMAL-ONLY REMOVED**] (1421)
44. (editorial or news or newspaper item).pt. (857,266)
45. 43 not 44 [**OPINION PIECES REMOVED**] (1388)
46. Systematic Review.pt. (222,586)
47. exp Systematic Reviews as Topic/ (10,119)
48. Meta Analysis.pt. (177,049)
49. exp Meta-Analysis as Topic/ (26,570)
50. (meta-analy* or metanaly* or metaanaly* or met analy* or integrative research or integrative review* or integrative overview* or research integration or research overview* or collaborative review*).tw,kw,kf. (269,949)
51. (systematic review* or systematic overview* or evidence-based review* or evidence-based overview* or (evidence adj3 (review* or overview*)) or meta-review* or meta-overview* or meta-synthes* or mapping review? or rapid review* or "review of reviews" or scoping review? or umbrella review? or technology assessment* or HTA or HTAs).tw,kw,kf. (356,504)
52. exp Technology Assessment, Biomedical/ (12,075)
53. (cochrane or health technology assessment or evidence report or systematic reviews).jw. (22,020)
54. Network Meta-Analysis/ (4624)
55. (network adj (MA or MAs)).tw,kw,kf. (17)
56. (NMA or NMAs or MTC or MTCs or MAIC or MAICs).tw,kw,kf. (9225)
57. indirect* compar*.tw,kw,kf. (2728)
58. (indirect treatment* adj1 compar*).tw,kw,kf. (452)
59. (mixed treatment* adj1 compar*).tw,kw,kf. (524)
60. (multiple treatment* adj1 compar*).tw,kw,kf. (223)
61. (multi-treatment* adj1 compar*).tw,kw,kf. (3)
62. simultaneous* compar*.tw,kw,kf. (1288)
63. mixed comparison?.tw,kw,kf. (44)
64. or/46–63 (543,322) [**SR FILTER**]
65. 45 and 64 [**IMMUNE CHECKPOINT INHIBITORS, TKIs, COMBINATION DRUG THERAPY - ENDOMETRIAL, RENAL CANCER - SRs etc**.] (128)
